# Single-emitter super-resolved imaging of radiative decay rate enhancement in dielectric gap nanoantennas

**DOI:** 10.1038/s41377-023-01349-2

**Published:** 2024-01-02

**Authors:** R. Margoth Córdova-Castro, Bart van Dam, Alberto Lauri, Stefan A. Maier, Riccardo Sapienza, Yannick De Wilde, Ignacio Izeddin, Valentina Krachmalnicoff

**Affiliations:** 1 Institut Langevin, ESPCI Paris, PSL University, CNRS, Paris, France; 2 The Blackett Laboratory, Department of Physics, Imperial College London, London, UK; 3 School of Physics and Astronomy, Monash University, Clayton, Victoria Australia; 4 Chair in Hybrid Nanosystems, Ludwig-Maximilians Universität München, Muenchen, Germany; 5 Present Address: Department of Physics, University of Ottawa, Ottawa, ON Canada

**Keywords:** Nanophotonics and plasmonics, Nanophotonics and plasmonics

## Abstract

High refractive index dielectric nanoantennas strongly modify the decay rate via the Purcell effect through the design of radiative channels. Due to their dielectric nature, the field is mainly confined inside the nanostructure and in the gap, which is hard to probe with scanning probe techniques. Here we use single-molecule fluorescence lifetime imaging microscopy (smFLIM) to map the decay rate enhancement in dielectric GaP nanoantenna dimers with a median localization precision of 14 nm. We measure, in the gap of the nanoantenna, decay rates that are almost 30 times larger than on a glass substrate. By comparing experimental results with numerical simulations we show that this large enhancement is essentially radiative, contrary to the case of plasmonic nanoantennas, and therefore has great potential for applications such as quantum optics and biosensing.

## Abstract (web summary)

Single-molecule fluorescence lifetime imaging microscopy (smFLIM) measures the radiative decay rate of single molecules in the near field of dielectric nanoantennas providing super-resolved information of the local density of states.

## Graphical abstract

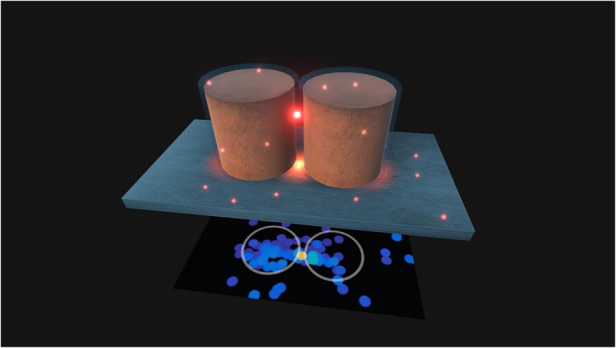

## Introduction

In recent years, plasmonic nanostructures have been extensively studied for a number of nanophotonic applications due to the capability to engineer large field enhancements at optical frequencies^1–4^, for example by nanoantenna dimers^5^, nanocones^6^, bowtie antennas, and nanocubes^7^. Strong confinement of the electric field at the hotspots of the nanoantenna enhances light-matter interaction for quantum emitters placed at these spots. Plasmons in metals have been extensively studied to modify the local density of states (LDOS) which is directly related to the probability of spontaneous light emission by a fluorescent emitter due to the strong field enhancement in metallic nanostructures^2,4,8–14^. However, one limitation inherent to the use of metals is their high optical absorption^15,16^. This brings a significant non-radiative contribution to the total decay rate, *Γ* = *Γ*_*r*_ + *Γ*_*nr*_, where *Γ*_*r*_ is the radiative and *Γ*_*nr*_ the non-radiative decay rate^11,17,18^. On the contrary, dielectric nanoantennas, while still offering all the advantages of tunability of their resonance frequencies and modal structures, can be free from such strong non-radiative contributions. Similar to plasmonic nanoantennas, resonant all-dielectric nanoantennas are able to convert propagating fields into localized near-fields, and they can exhibit far-field optical cross sections several times larger than their physical dimensions^19–25^. Moreover, dielectric nanoantennas support Mie modes which can be both magnetic and electric in the visible spectral range^24–27^. Since for strong Mie resonances a large refractive index contrast between the dielectric material and the surrounding medium is required, particles made out of materials such as AlGaAs, Ge and Si are often exploited^28–30^. Another promising material is gallium phosphide (GaP). It has an associated bandgap wavelength as small as 550 nm with the real part of refractive index ~3.3 and a very small imaginary part of refractive index, making it interesting for low-loss nanophotonics antennas in the optical regime. GaP nanantennas have allowed efficient second harmonic generation (SHG)^31,32^, all-optical switching and integrated wave-guides, capable of covering almost the entire visible range with negligible losses. It has been shown that nanoantennas composed of two GaP nanodisks separated by a nanosized gap can provide confined optical modes with strongly reduced mode volumes^33–35^. Moreover the presence of electric dipolar (ED) and magnetic dipolar (MD) resonances in the visible spectral range makes this structure attractive for nanophotonic devices^36^. Previous reports have highlighted the low non-radiative losses of GaP nanostructures for excitation energies below their band gap^20,21,33,34,36^.

The Mie resonances of dielectric antennas can also be used to control the fluorescence of emitters in their proximity. Strong fluorescence enhancement and lifetime reduction by more than 20-fold have been shown experimentally by ensemble measurements of dye molecules randomly distributed around GaP dimer antennas^33^, and 10^3^-fold photoluminescence enhancement for atomically thin semiconductors on GaP nanoantennas^34^. However, these results report the global response of the antenna and lack spatial information at the relevant nanometer scale and at the single fluorescent emitter level.

In this paper, we use single molecule fluorescence lifetime imaging microscopy (smFLIM)^37–39^ to quantify the LDOS modification induced by GaP nanodimers via the decay rate modification of individual fluorescent molecules placed around the nanostructure. With a median localization precision of 14 nm, we show that the antenna induces around 30-fold decay rate enhancement in the 20 nm gap with respect to the decay rate measured on the substrate. Numerical simulations demonstrate the radiative origin of the enhancement. The study of the point spread function’s (PSF) shape and the comparison with numerical simulations show that molecules in the gap are mostly exempt from a lateral mislocalization error, or mirage effect, due to the presence of the nanostructure^38,40^, which corroborates our findings that molecules with the highest decay rate enhancements are physically located in the gap.

## Results

**Fig. 1:**
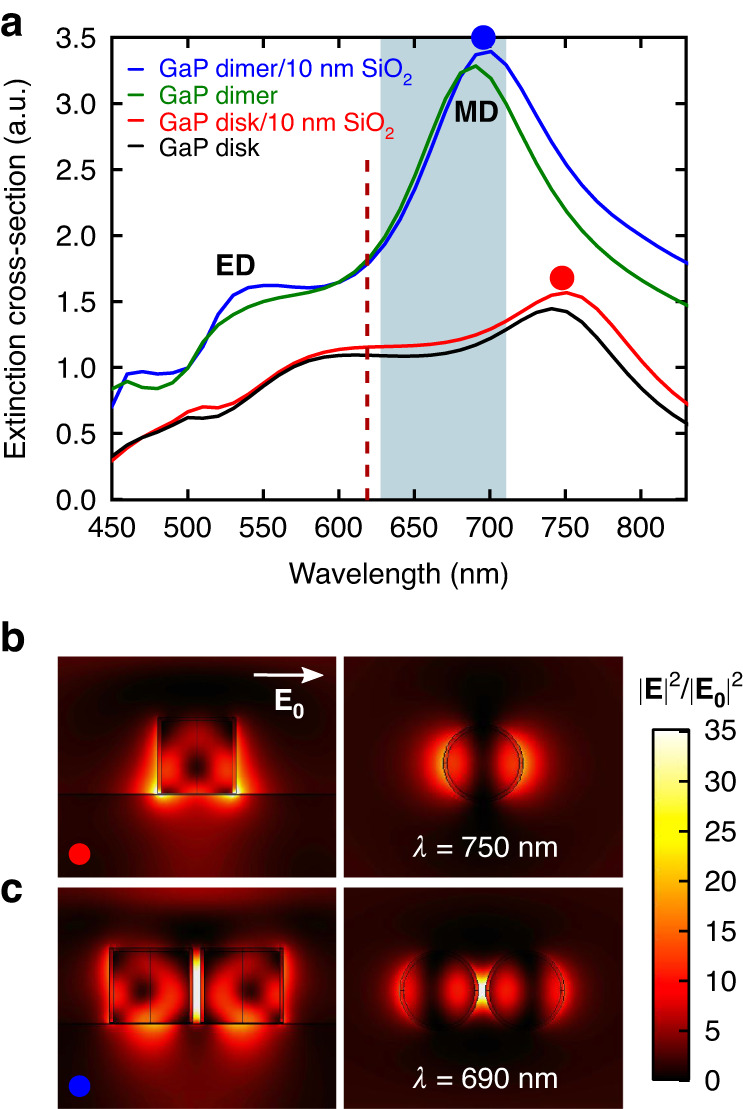
Simulated photonic properties of a GaP monomer and dimer. **a** Simulated extinction cross section for a GaP monomer with a 200 nm diameter and a height of 200 nm and a GaP dimer with a 40 nm gap with and without a 10 nm SiO_2_ layer around the GaP disks. The red dashed line indicates the excitation wavelength of the laser and the blue shadowed region corresponds to the emission spectrum of the fluorescent molecules used for this work. **b**, **c** Near field intensity map calculated for an excitation wavelength of 750 nm for the monomer and 690 nm for the dimer coated with a 10 nm SiO_2_ layer. Simulations are performed by illuminating the structure with a plane wave polarized along the dimer axis. Data are normalized with respect to the incident intensity. For the sake of completeness, simulations driven at different wavelengths for both the monomer and the dimer, with and without the SiO_2_ layer are reported in Figs S1 and S2

The simulated photonic properties of a GaP dimer, formed by two disks of 200 nm height and 200 nm diameter separated by a gap of 40 nm, on a glass substrate are shown in Fig. 1. The antenna is excited with a plane wave polarized along the dimer axis propagating normally to the glass substrate. For comparison, the photonic properties of a monomer consisting of a single GaP disk of 200 nm height and 200 nm diameter are shown in the same figure.

The Mie resonances of the disks give rise to peaks in the extinction cross-section spectra. Interestingly, the electric and magnetic resonances which are barely visible at a wavelength of 575 nm and 740 nm in the extinction cross-section of the monomer (Fig. 1a) are sharpened and blue-shifted for the dimer antenna. We notice that at these dimensions, the extinction is dominated by scattering while absorption is almost zero due to the negligible imaginary part of the dielectric function of GaP. The introduction of a 10 nm thick SiO_2_ layer around the disk results in an enhancement of the extinction cross-section and a spectral shift of about 10 nm for both the monomer and the dimer. In this study we focus on SiO_2_-capped GaP nanoantennas.

Figure 1b, c reports the near-field maps of the electric-field intensity enhancement with respect to the incident field, (|E|/|E_0_|)^2^, at the resonant frequency for the MD mode, for a SiO_2_-capped GaP monomer and dimer respectively. As it can be noticed by comparing the results for the monomer and the dimer, the presence of the second GaP disk enhances the hotspot generated by the monomer at the disk base and generates a strong hotspot in the gap of the nanoantenna. Here, the intensity is enhanced by more than a factor 35 with respect to the incident intensity.

Due to the mainly radiative character of the GaP dimer, the presence of such an intensity hotspot in the gap enables inferring its presence at the same location in the LDOS. Directly revealing the presence of such a hotspot in the gap of the GaP dimer is a challenge due to the dimensions of the nanostructure. The gap between the disks is narrow and deep which makes it difficult to controllably place a single fluorescent probe inside of it. A good candidate for probing the interaction of single molecules within the GaP dimer, is smFLIM (single-molecule Fluorescence Lifetime Imaging Microscopy). smFLIM combines Single Molecule Localization Microscopy (SMLM)^41–43^ with single-molecule fluorescence lifetime measurements^37–39,44^. This enables super-resolved imaging of the decay rate modification of single molecules as a function of their position at a distance of a few nanometers from the nanostructure’s surface^37,38^. Since the decay rate of a fluorophore is directly related to the LDOS of the environment, this enables direct measurement of the LDOS at the location of each emitter at its emission wavelength^13^. The sample is densely labeled with small fluorescent molecules that can easily diffuse and bind to the surface inside the narrow gap, while still obtaining single-molecule detection. This represents an advantage of smFLIM with respect to other techniques such as fluorescent near-field scanning probes^13^, fluorescent emitters flowing in a microfluidic channel^45^, or moving thanks to biomolecular motors^46^. While these latter techniques offer the advantage of an a priori knowledge of the position of the emitter, measurements inside narrow gaps are challenging due to either the relative size of the probe or poor spatial sampling control.

We labeled our samples with Abberior Cage 635 fluorophores which become photo-active upon illumination with a continuous wave UV laser. To enable single-molecule detections, its intensity is set so that the probability of having more than one active molecule at the same time in a diffraction limited spot is negligible. Active molecules are excited with a pulsed laser at a wavelength of 625 nm, with a repetition rate of 40 MHz. The excitation laser is linearly polarized parallel to the dimer axis to primarily excite molecules aligned with the dimer. The sample is excited through an oil immersion microscope objective (NA = 1.49) in a wide-field total internal reflection geometry. Fluorescence photons are filtered between 633 nm and 700 nm where the field enhancement in the gap is maximum (see Fig. 1a and Fig. S3), then collected through the same objective and split into two parts. Half of the photons are detected by an EM-CCD camera for PSF imaging and position estimation. The other half of the photons are detected by an 8×1 Single Photon Avalanche Diode (SPAD) array combined with a Time-Correlated Single Photon Counting (TCSPC) system for decay rate measurement. Each pixel of the SPAD array is conjugated with a region of around 1 µm^2^ on the sample plane and the density of photo-active molecules is set by the activation laser intensity to ensure that only a single molecule is detected at a given time on a SPAD in the array while it is simultaneously imaged with the EMCCD camera. A sketch of the experimental setup is reported in Fig. S4. The combination of a pulsed excitation laser and the customized TCSPC^47^ system enables one to record the photon arrival times of the fluorescence photons emitted by each molecule and to retrieve the decay histogram with a few picosecond resolution. The total decay rate *Γ* is then measured by fitting the decay histogram with a mono-exponential decaying function convoluted with the instrument response function (IRF) of the setup. The association of the measured decay rate and the position allows us to reconstruct a super-resolved decay rate map which is related to the LDOS on the sample surface. The single-molecules’ position on the sample plane is determined with a median precision of 14 nm by fitting the single-molecule PSFs with a two-dimensional Gaussian function. A more detailed description about the sample preparation and the setup configuration as well as the data analysis can be found in the Methods section.

We studied two different sets of GaP nanoantennas of 200 nm in height and 200 nm in diameter with a gap between the disks of 20 and 40 nm respectively. Such antennas are fabricated by electron-beam lithography and reactive ion etching (more fabrication details can be found in the Methods section). A 10 nm SiO_2_ layer was deposited around the nanostructure by atomic layer deposition (ALD) to enhance the optical properties and to allow the uniform functionalization of single molecules on the nanostructure surface, inside the gap and on the glass substrate. This enables a direct comparison of the decay rate enhancement in absence and presence of the nanostructure.

**Fig. 2:**
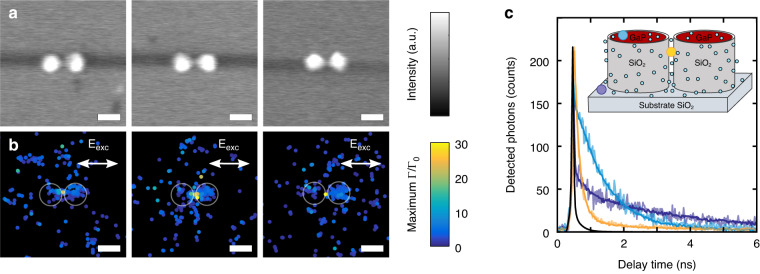
smFLIM image of the decay rate enhancement induced by GaP dimers. **a** Scanning electron microscopy images of three GaP dimers with a diameter of 200 nm and gaps of 20, 20, and 40 nm respectively. The scale bar represents 200 nm. **b** Single molecule decay rate enhancement maps measured for the same dimers as in the corresponding panels in (**a**). The maximum value of the decay rate enhancement measured at each position is shown, normalized to the decay rate of molecules on glass. Black areas indicate positions where no events are localized. White circles indicate the position of the disks. The typical localization precision of the single fluorescent molecules is 14 nm. **c** Typical fluorescence decay curves of single molecules localized at different positions around the third dimer: On top of a disks (blue, *Γ* = 1.2 ns^−1^), in the gap of the dimer (orange, *Γ* = 8.7 ns^−1^) and far away from the dimer (purple, *Γ* = 0.3 ns^−1^). The decay rate is extracted by fitting the decay histograms with a mono-exponential decay convoluted with the instrument response function of the setup (IRF, black), taking into account the background signal

The super-resolved decay rate images, normalized by the value measured on glass, for three GaP dimers are shown together with the corresponding scanning electron microscopy (SEM) images in Fig. 2a, b. Each decay rate image is reconstructed from ~250 super-localized single-molecule events. The comparison with the SEM images shows that the highest decay rate is observed in the gap of the dimer with a measured decay rate enhancement up to ~20–30 with respect to the one measured on glass far away from the GaP dimer (between 0.2 and 0.4 ns^−1^). The strongly enhanced decay rate for molecules placed in the gap indicates a strongly enhanced LDOS as is expected since the calculated field enhancement is higher than on glass (see Fig. 1). Most detections are observed on the top of the disks, with decay rates ranging between 2 and 7 ns^−1^ while events detected further away from the dimer are characterized by decay rates between 0.3 and 1 ns^−1^, similar to the decay rate of molecules on glass in the absence of the dimer. Typical decay rate histograms measured at different positions around the dimer (in the gap, on top of one disk, and glass) are shown in Fig. 2c.

## Discussion

**Fig. 3:**
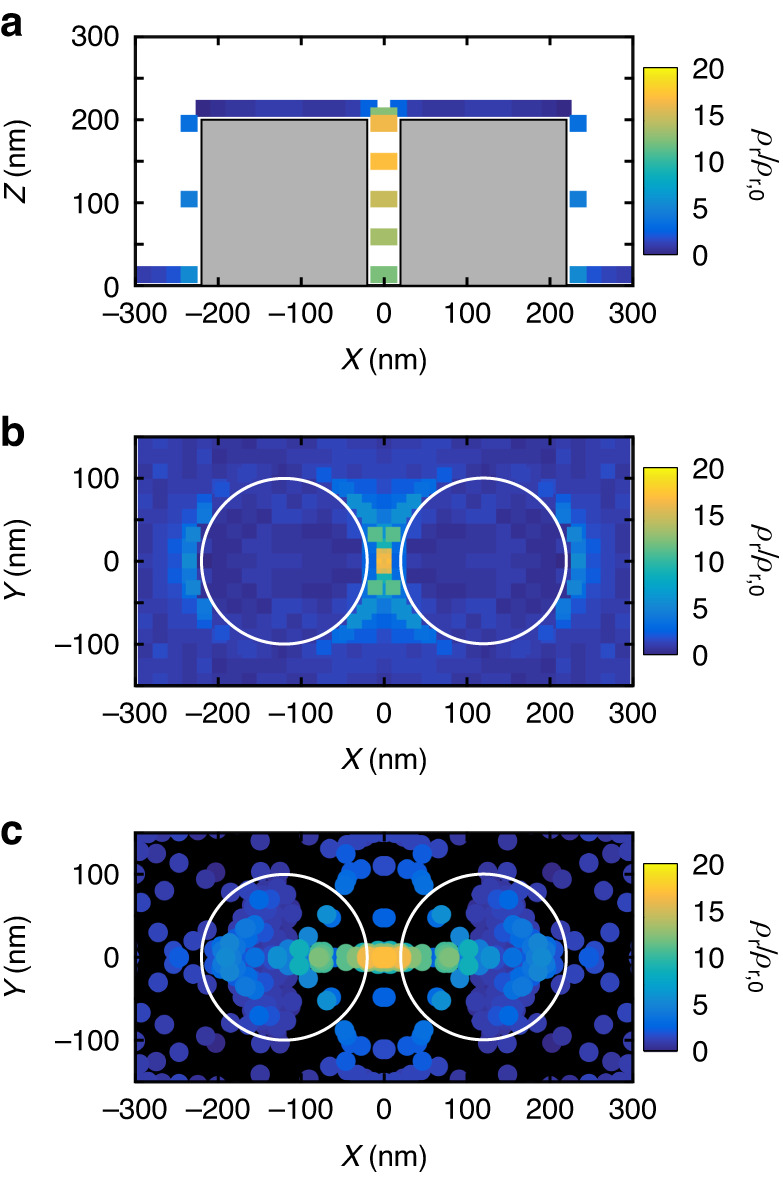
Simulated radiative LDOS enhancement maps around the GaP dimer. **a** Side view of the simulated LDOS along the dimer axis (Y = 0). Gray shaded area indicates the position of the GaP pillars. **b** Top view of the simulated LDOS for dipoles positioned at a distance of 4 nm from the GaP pillars, normalized by the LDOS of a dipole on glass. All the dipoles are oriented parallel to the X axis. **c** Simulated LDOS enhancement plotted against the apparent dipole position. The apparent position is obtained by fitting the dipole’s recorded point spread function with a 2D Gaussian function. The white circles indicate the edges of the GaP pillars

Dielectric nanoantennas like the GaP disk dimers are interesting for their inherent radiative decay rate enhancement associated with low non-radiative losses. This becomes evident when comparing the numerically simulated radiative and total LDOS and enables to state that the largest contribution to the measured decay rate enhancement is the radiative one. Using Finite-Difference Time–Domain (FDTD) simulations we extracted the power radiated from single electric dipoles coupled to the nanoantenna located at different positions around a dimer with a gap of 40 nm. The resulting map of the radiative LDOS for dipoles with their dipole moment oriented along the dimer axis (X-axis) is shown in Fig. 3a, b. A significant enhancement of the LDOS is found for dipoles located inside the gap with, depending on the z-position of the dipole (Fig. 3a), an enhancement spanning from ~10 to 20. This variation is expected from the field enhancement distribution along the gap which is not uniform, as shown in Fig. 1. A smaller enhancement (about a factor of 6) is also seen for dipoles placed on the other side of the dimer. Correspondingly, simulations reported in Fig. 1b show a small enhancement of the electric field on the side of the dimer. The total calculated LDOS (Fig. S6) shows an almost identical map to the radiative LDOS, indicating that the enhancement is mainly due to radiative modes. This is consistent with negligible losses in GaP related to the almost zero imaginary part (from 0 to 0.027) of the refractive index in the visible range^48^. This demonstrates that the measured decay rate enhancement in Fig. 2 is also radiative. We notice that the difference between the values of the simulated LDOS map and the experimental one corresponds to the inherent imperfection of the fabricated structure, as for gap definition and cleanness and the presence of the SiO_2_ layer in the gap. Numerical calculations show that the maximum LDOS enhancement strongly depends on the position of the dipole in the gap (Fig. 3a). Such LDOS variation in the gap could enable the determination of the z position of the molecule in the gap by its comparison to the measurement of the decay rate^38^. However, the deepness of the gap and its narrowness hinder the presence of many molecules in the gap, limiting the number of available measurements and therefore preventing such measurement in this specific case.

For completeness we also performed simulations for dipoles with their moment oriented along the Y and Z axis, (***µ*** = *µ*_***y***_ and ***µ*** = *µ*_***z***_). As shown in Fig. S5, those dipoles only show a small or no enhancement of the radiated power because the Mie modes excited in the disks are not interacting with the emitter, as they are mainly distributed inside the dielectric disks. Consequently, the field enhancement is not significant outside the nanostructure and the power emitted by the dipole is slightly or not modified^33^. This is in strong contrast with dipoles with their dipole moment aligned with the dimer axis (***µ*** = *µ*_***x***_). To excite and detect primarily those dipoles orientations for which we expect the highest LDOS enhancement in our experiments, we therefore choose to excite the molecules with a p-polarized plane wave along the dimer axis.

A key assumption underlying most single-molecule microscopy analysis techniques is that the center of the PSF of a single molecule determines its true position on the sample^39^. While this assumption holds for an isotropic point emitter in an isotropic environment, single molecules emission is known to depend on the orientation of the molecular dipole moment and on the environment, which results in a deformation of the three-dimensional PSF^38^. Notably, when the molecule is placed in the near field of a nanostructure, the coupled radiations might result in a strong PSF deformation and an associated mislocalization of the emitter^49^. Previous reports have noted significant localization errors up to 200 nm, when employing typical estimators of the molecular position from a detected image such as centroid calculation or fitting to a two-dimensional (2D) Gaussian function^38,40^.

To study mislocalization effects in the case of the GaP nanodimer, we simulated with FDTD the far-field emission pattern reconstructed in real space as collected by a numerical aperture of 1.49. As in our experiments, we used a 2D normal distribution to estimate the center of mass of the simulated PSFs and used the apparent dipole positions to plot the LDOS map in Fig. 3c. The difference with the real positions shown in Fig. 3b is notable. Interestingly, the spatial distribution of the detected dipoles is strongly modified, showing that dipoles physically located on the top of the disks are mainly detected in the central region of the disks, leaving zones with apparently no detections. Similarly, dipoles located between the disks will be detected in the middle of the gap, and the LDOS gradient that appears between the disks in the real-dipole-position image completely disappears in the apparent-dipole-position one. This observation is in excellent agreement with the experimental observations proving that the spatial inhomogeneity of detections is not due to a labeling issue but mainly to the mirage effect.

Moreover, the highest enhancement of the LDOS is indeed obtained for dipoles with an apparent and real position inside the gap, meaning that the PSF of such molecules does not undergo significant distortion. On the contrary, dipoles that are set on the side of the dimer and that experience a LDOS enhancement of about a factor of 10 are located on the top of the dimer by the 2D PSF’s Gaussian fit. This is consistent with the experimental observations, in which molecules with a decay rate enhancement of a factor of ~10 are detected on the top of the GaP pillar.

**Fig. 4:**
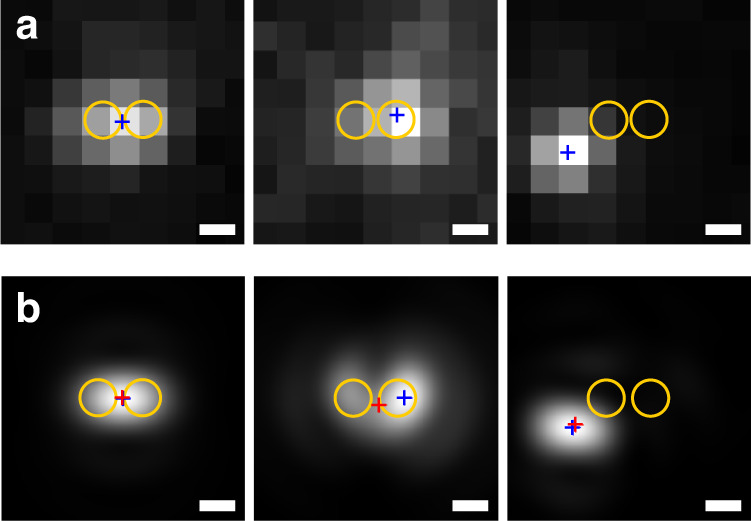
Experimental and simulated PSF deformation induced by the presence of the GaP dimer. **a** Measured point spread function (PSF) for the single-molecule events shown in Fig. 1c. From left to right: Molecule located in the gap, on top of a disk, far away from the disk. The orange circles indicate the position of the disks; the blue cross indicates the apparent position of the molecule, as estimated from the recorded PSF. The scale bars represent 200 nm. **b** Simulated PSFs for dipoles with similar decay rate and apparent position as those in (**a**). The red crosses indicate the real dipole position used for each simulation

These findings are corroborated by the direct observations of experimental and simulated PSFs. Typical PSFs obtained experimentally and numerically are shown in Fig. 4a, b respectively for three different positions of the molecules on the sample, i.e., in the gap, on the side of one disk, and the glass substrate. The yellow circles represent the dimer, the blue crosses represent the apparent position as estimated from the fit of the PSF, while the red crosses represent the real position of the dipoles that generated such PSF. We can notice that the PSF of the molecules inside the gap are slightly elongated along the dimer axis for both the experiment and the simulation, but centered at the actual dipole position. For a molecule that is detected on the top of a disk, the mislocalization can be as large as 150 nm, while for molecules located on the substrate, 100 nm away from the dimer, the mislocalization error is only a few nanometers.

To summarize, we show that it is possible to image the spatial dependent interaction of single fluorescent emitters with GaP dielectric gap nanoantennas. Using smFLIM, we localize single emitter positions with a median localization precision of 14 nm and directly measure their Purcell enhancement, as high as 30-fold for emitters in the gap of the antenna. A thorough numerical study of the PSF deformation and its comparison with experimental findings show that the position of fluorophores is affected by only a few nanometers mislocalization due to interaction with the nanostructure. This corroborates the experimental observation that emitters which are physically in the gap experience the highest decay rate enhancement. We show an excellent agreement between the simulated and experimentally measured spatial variation of the radiative LDOS enhancement when the apparent rather than the real position of single emitters is considered. A radiative-only fluorescence enhancement of a single molecule with no optical loss as in dielectric nano-antenna has great potential for applications such as quantum optics and biosensing.

## Materials and methods

### Nanoantenna fabrication

The nanoantennas are fabricated by electron-beam lithography of a sputtered GaP layer on glass, and subsequential etching by reactive ion etching (RIE)^33,34^.

### Single molecule sample preparation

After fabrication, the sample was cleaned by sonication in acetone during 20 min, rinsed with demineralized water, dried and further cleaned by an Ozone treatment for ~20 min. The cleaned sample was then functionalized with a layer of bovine serum albumin (BSA, Sigma Aldrich) by introducing a solution of BSA in phosphate-buffered saline (PBS, Sigma Aldrich) at 0.9 mg mL^−1^ for 20 min. The BSA was labeled with fluorophores by adding a 40:5:1 mixture of PBS, sodium bicarbonate solution (7.5%v, Sigma Aldrich) and photoactivatable Abberior CAGE 635 (NHS ester conjugate) in Dimethylsulfoxide at 0.6 mg mL^−1^ (DMSO, Sigma Aldrich) for 30 min. Polystyrene fluorescent beads (Crimson FluoSpheres 200 nm, ThermoFisher Scientific) were also dispersed in PBS and used as fiducial markers. The sample was rinsed with PBS between each step. In the final step the sample was rinsed with demineralized water and dried by spin-coating at 3000 rpm.

### Optical setup

smFLIM was carried out on an inverted microscope (X71, Olympus) equipped with a NA = 1.49 microscope objective (UApoN 100X, Olympus) in a wide-field total internal reflection geometry. The fluorescent molecules were excited through the microscope objective with a super continuum pulsed laser (NKT Photonics) coupled to an acousto-optic tunable filter (SuperK Select, NKT Photonics) transmitting a narrow band around a wavelength of 625 nm. For excitation we used a repetition rate of 40 MHz. The excitation polarization was set parallel to the axis of the dimers. Photoactivation of the molecules was achieved with a continuous wave 405 nm laser diode (Oxxius) coupled into the same excitation path as the pulsed laser.

Single-molecule fluorescence was collected by the same objective lens and filtered through a long-pass filter (BLP01-633R, Semrock). Using a 50:50 beam-splitter, half of the collected photons were imaged on an EMCCD camera (iXon Ultra, Andor), while the remaining half was guided on a linear array of single-photon avalanche diodes (SPADs) connected to a time-correlated single photon counting module (TCSPC)^47^. During the measurements, we corrected for sample drift by imaging a nearby fiducial marker and maintaining it at fixed position and focal plane using a piezoelectric stage (PInano XYZ, Physik Instrumente). Prior to the measurements, the position of each SPAD detector with respect to the CCD field of view (FOV) was calibrated by scanning a fluorescent bead in the sample plane.

### Data analysis

Localization of single-molecule emission was performed by fitting each point spread function (PSF) with a 2D Gaussian function using the ThunderSTORM^50^ plugin in ImageJ. The results of the ThunderSTORM algorithm provide the coordinates for each detected single fluorophore with the error associated with its position. This localization precision is ultimately dependent on the number of photons detected per PSF. The best estimate of the position is given by the average of the positions of the individual detected photons, with an error given by common statistical formula for the standard error on the mean, $\left\langle {\left({{\rm{\sigma }}}_{{xy}}\right)}^{2}\right\rangle =\frac{{s}^{2}+{a}^{2}/12}{N}+\frac{8\pi {s}^{4}{b}^{2}}{{a}^{2}+{N}^{2}}$, where ${\rm{\sigma }}$_xy_ is the localization precision, *s* is the standard deviation of the PSF (Gaussian or otherwise), *a* is the size of the pixel, *b* is the background noise, and *N* is the number of photons collected. The obtained location estimates were further processed using a home-built script, in which single-molecule detections that appear in multiple consecutive frames were first merged and then associated with the time-resolved data recorded by the TCSPC. Frames in which multiple detections coincide with the FOV of a single SPAD were omitted from further data analysis to prevent the mixing of decay rate information of multiple molecules. The typical localization precision (σ_xy_ ~ 14 nm) was estimated by the median localization precision of all the events that were detected on both the SPAD and EMCCD. Decay rate estimates were obtained by fitting the decay rate histogram with a mono-exponential decaying function convoluted with the instrument response function (IRF), allowing a small time-shift between the IRF and the recorded decay rate histogram. A background signal was added to the fitting model to correct for background luminescence and dark counts of the SPAD. Fitting optimization was performed by using a maximum likelihood method, assuming a Poissonian distribution of the counts recorded in each time channel.

From the position and decay rate of each correlated single-molecule event, a decay rate map can be reconstructed. Here we choose to plot the maximum decay rate enhancement, by taking the maximum decay rate obtained at each position *Γ*_*max*_(*x,y*), normalized to the average decay rate of molecules on the substrate in the absence of the GaP dimers (*Γ*_*0*_ ~ 0.3 ns^−1^). More information on the smFLIM measurements can be found in our previous works^37,38^.

### Numerical simulations

We solve Maxwell’s equations using a finite-difference time domain FDTD commercial solver (Lumerical Solutions, Inc., Vancouver) for Purcell factor calculations. We considered the three principal Cartesian orientations for radiating dipole calculations at a distance of 4 nm to the substrate and the dimers surface, reproducing the experimental condition of the fluorophore biotin linker. We evaluate the total LDOS by integrating the total power radiated by both the dipole and the nanostructure and normalizing it to the power of a dipole on glass. For the radiative part of the LDOS, we integrated the power radiated by the electric dipole alone, emitted from a small bounding spherical domain of 5 nm radius with a fine mesh around the dipole. For imaging, we simulated the far-field emission pattern of the dipoles reconstructed in real space as collected by a 1.49 numerical aperture lens. The resulting point spread function and total and radiated power values are exported from Lumerical and analyzed in MATLAB.

We used a 2D normal distribution to estimate the center of mass of the obtained PSFs, as in the analysis of the experimental data to compare the apparent dipole position obtained and the simulated dipole position.

The extinction cross-section and the near-field intensity distribution were calculated using the commercially available finite-element method solver COMSOL Multiphysics with the RF module. The simulation region was restricted by perfectly matched layer boundaries to imitate antennas in an infinite space. Experimental values of the dielectric function of crystalline GaP were taken from Aspnes et al.^48^

### Supplementary information

Supplementary Information for: “Single-emitter super-resolved imaging of radiative decay rate enhancement in dielectric gap nanoantennas”
