# Supplementary material for: Single-emitter super-resolved imaging of radiative decay rate enhancement in dielectric gap nanoantennas
Source: Light Sci Appl. 2024 Jan 2;13:7. doi: 10.1038/s41377-023-01349-2 (PMC10761855; doi:10.1038/s41377-023-01349-2)
Supplement: Supplementary file 1 — Supplementary Information for: “Single-emitter super-resolved imaging of radiative decay rate enhancement in dielectric gap nanoantennas” [file 41377_2023_1349_MOESM1_ESM.pdf]

SUPPLEMENTARY INFORMATION

Supplementary Information for: "Single-emitter super-resolved imaging of radiative decay rate enhancement in dielectric gap nanoantennas"

R. Margoth Córdova-Castro,[1†‡] Bart van Dam,[1‡] Alberto Lauri,[2] Stefan A. Maier,[2, 3, 4] Riccardo Sapienza,[2] Yannick De Wilde,[1] Ignacio Izeddin,[1*] and Valentina Krachmalnicoff[1*]

[1]Institut Langevin, ESPCI Paris, PSL University, CNRS, 75005 Paris, France
[2] The Blackett Laboratory, Department of Physics, Imperial College London, London SW7 2BW, United Kingdom
[3] School of Physics and Astronomy, Monash University, Clayton Victoria 3800, Australia
[4] Chair in Hybrid Nanosystems, Ludwig-Maximilians Universität München, 80539 Muenchen, Germany
† Current address: Department of Physics, University of Ottawa, Ottawa ON K1N 6N5, Canada.

‡ Equal contribution
[*]Corresponding authors: ignacio.izeddin@espci.fr and valentina.krachmalnicoff@espci.fr

1. Excitation intensity enhancement simulations

We report FDTD simulations for the calculation of the field intensity enhancement with respect to the incident intensity for different wavelengths. The structure is either a GaP monomer or a dimer and is excited with a p-polarized plane wave impinging perpendicularly to the structure. In the case of the dimer, the polarization is set parallel to the dimer axis.

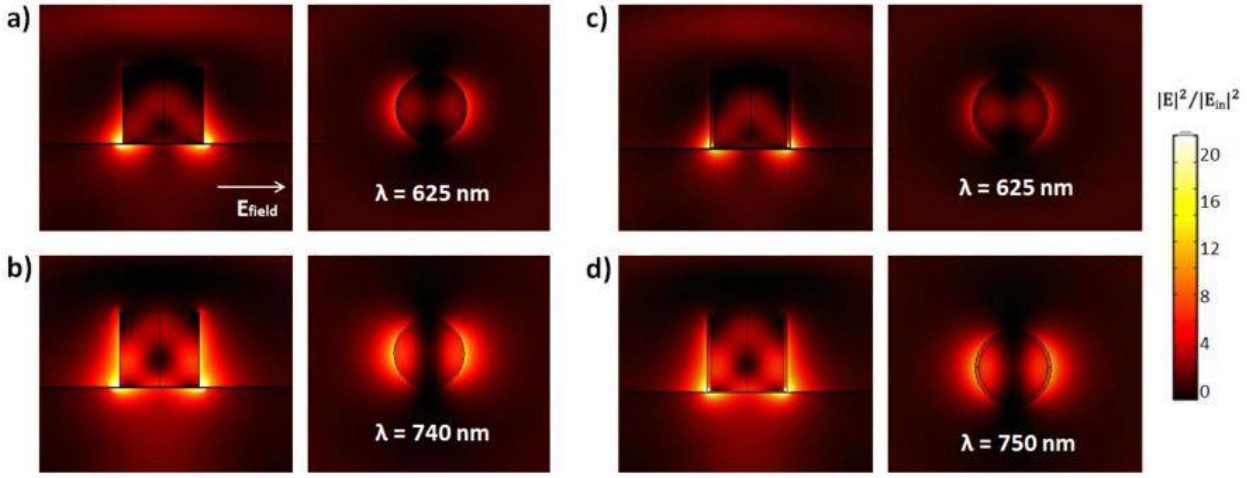

Figure S1. *Calculations of the normalized near-field intensity map (a,c) for the illumination wavelength of the experiment 625 nm and (b,d) for the maximum of the magnetic resonance at 750 nm for the GaP pillar with 200 nm diameter and 200 nm height with (c,d) and without (a,b) the 10 nm SiO_2 layer. Illuminated with a p-polarized plane wave as illustrated by the arrow in a).*

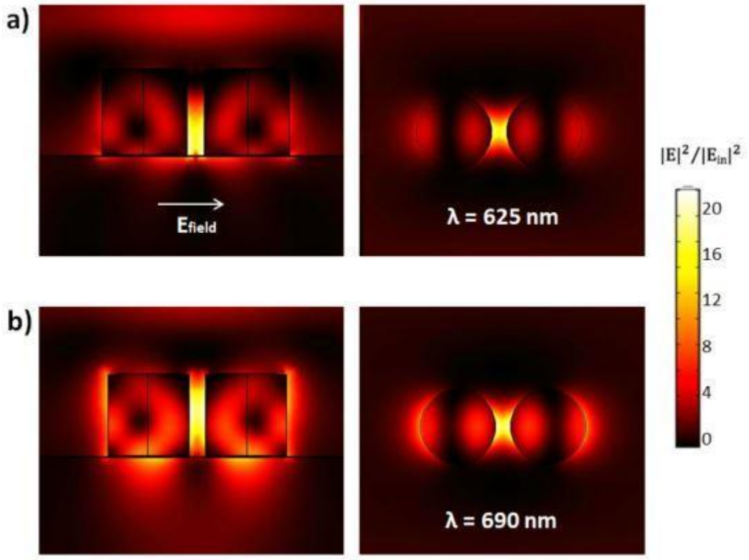

Figure S2. *Calculations of the normalized near-field intensity map (a) for the illumination wavelength of the experiment 625 nm and (b) for the maximum of the magnetic resonance at 690 nm for the GaP dimer. Each disk has a diameter of 200 nm and a height of 200 nm. The two disks are separated by a 40 nm gap. The structure is illuminated with a p-polarized plane wave with the incoming electric field oriented along the dimer axis.*

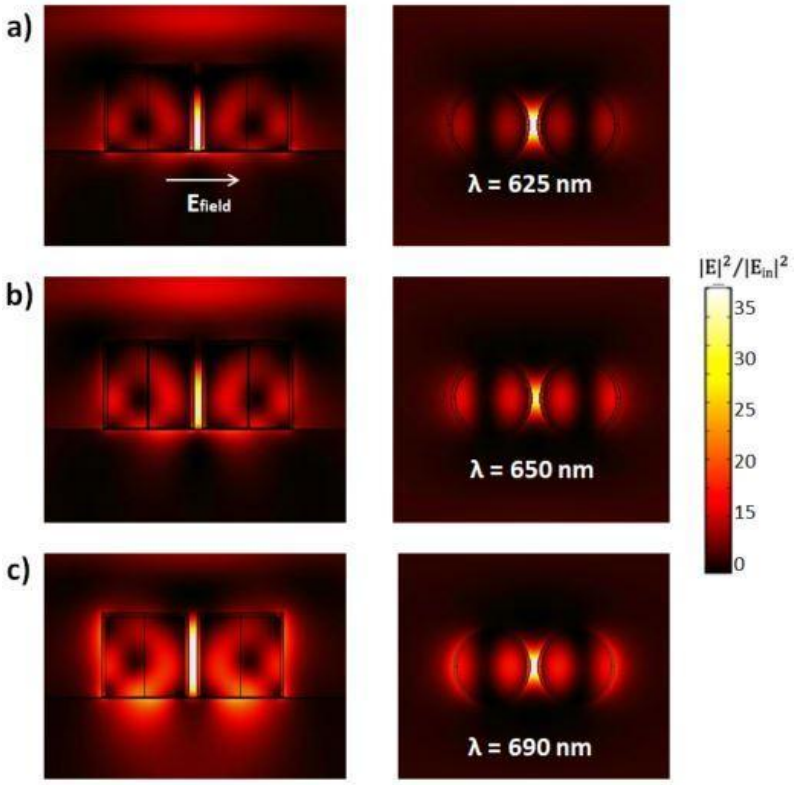

Figure S3. *Calculations of the normalized near-field intensity map (a) for the illumination wavelength of the experiment 625 nm, (b,c) for wavelength in the area of detection 650 nm and (c) for the maximum of the magnetic resonance at 690 nm for the GaP dimer with 200 nm diameter and 200 high separated by a 40 nm gap capped with a 10 nm* SiO_2 *layer. The structure is illuminated with a p- polarized plane wave oriented along the dimer axis. The enhancement shown in Figure S3a is a combination of the ED and MD resonances.*

2. smFLIM experimental setup

The smFLIM experimental setup is reported in Figure S4. The principle of the smFLIM approach is also sketched.

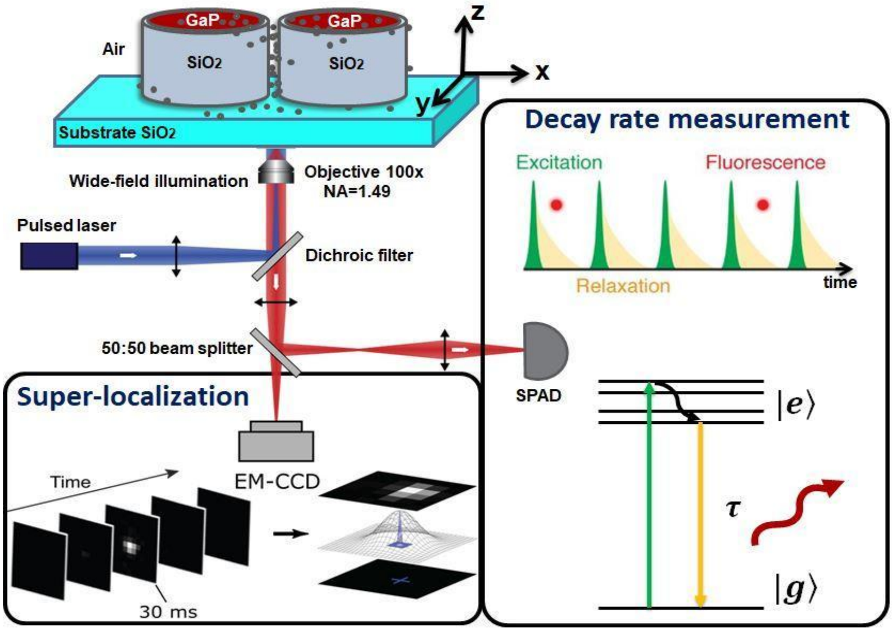

Figure S4. *Experimental setup and sketch of the smFLIM approach. The sample is excited in wide-field by a pulsed laser, through the substrate (a glass coverslip). Fluorescence photons are collected through a high NA objective and are split into two paths by a 50:50 beam-splitter. Half of the photons are detected by an EM-CCD camera and are used to image the PSF of each emitting molecule for its localization. The other half of the photons are detected on a SPAD array with a TCSPC system for the measurement of the lifetime of each emitting molecule.*

3. LDOS enhancement simulations

We report on the simulated LDOS enhancement for dipoles located at a distance of 4 nm from the dimer surface.

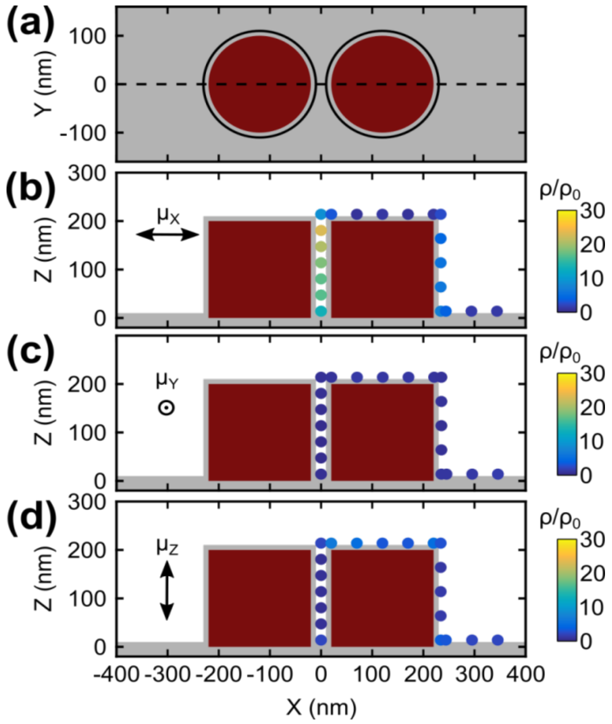

Figure S5. *Simulated LDOS enhancement for different positions of the dipole around a GaP dimer: (a) Schematic top view with the dashed line indicating the Y position of simulated dipoles. Each pillar has a diameter of 200 nm and a height of 200 nm and is coated with 10 nm of SiO2. (b-d) Simulated LDOS enhancement (side view) for dipoles with the dipole moment oriented along the X, Y and Z axis respectively. The LDOS is normalized to the value for a dipole in air.*

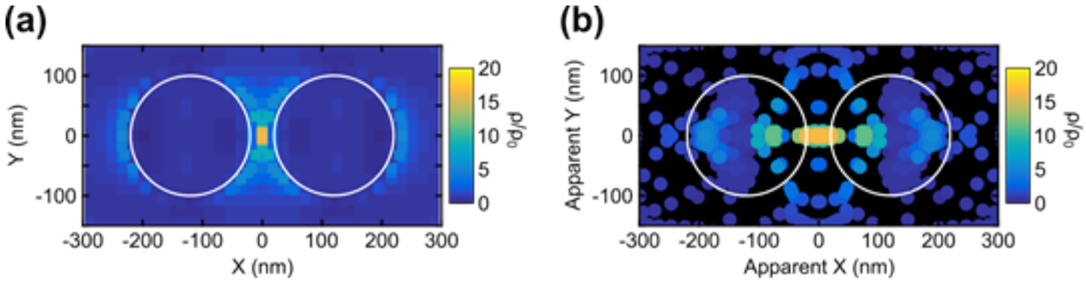

Figure S6. *Simulated total LDOS enhancement maps around the GaP dimer: (a) Top view of the simulated LDOS for dipoles positioned at a distance of 4 nm from the GaP pillars surface, normalized by the LDOS of a dipole on glass. All the dipoles are oriented parallel to the X axis. (b) Simulated LDOS enhancement plotted against the apparent dipole position. The apparent position is obtained by fitting the dipole's recorded PSF with a 2D Gaussian function. The white circles indicate the edges of the GaP pillars.*
